# Free Chlorine and Peroxynitrite Alter the Capsid Structure of Human Norovirus GII.4 and Its Capacity to Bind Histo-Blood Group Antigens

**DOI:** 10.3389/fmicb.2021.662764

**Published:** 2021-04-13

**Authors:** Manon Chassaing, Guillaume Bastin, Maëlle Robin, Didier Majou, Gaël Belliot, Alexis de Rougemont, Nicolas Boudaud, Christophe Gantzer

**Affiliations:** ^1^Food Safety Department, ACTALIA, Saint-Lô, France; ^2^Université de Lorraine, CNRS, LCPME, Nancy, France; ^3^ACTIA, Paris, France; ^4^National Reference Centre for Gastroenteritis Viruses, Laboratory of Virology, University Hospital of Dijon, Dijon, France; ^5^UMR PAM A 02.102 Procédés Alimentaires et Microbiologiques, Université de Bourgogne Franche-Comté/AgroSup Dijon, Dijon, France

**Keywords:** norovirus, virus-like particles, viral protein, histo-blood group antigens, free chlorine, peroxynitrite

## Abstract

Human noroviruses (HuNoVs) are one of the leading causes of acute gastroenteritis worldwide. HuNoVs are frequently detected in water and foodstuffs. Free chlorine and peroxynitrite (ONOO^−^) are two oxidants commonly encountered by HuNoVs in humans or in the environment during their natural life cycle. In this study, we defined the effects of these two oxidants on GII.4 HuNoVs and GII.4 virus-like particles (VLPs). The impact on the capsid structure, the major capsid protein VP1 and the ability of the viral capsid to bind to histo-blood group antigens (HBGAs) following oxidative treatments were analyzed. HBGAs are attachment factors that promote HuNoV infection in human hosts. Overall, our results indicate that free chlorine acts on regions involved in the stabilization of VP1 dimers in VLPs and affects their ability to bind to HBGAs. These effects were confirmed in purified HuNoVs. Some VP1 cross-links also take place after free chlorine treatment, albeit to a lesser extent. Not only ONOO^−^ mainly produced VP1 cross-links but can also dissociate VLPs depending on the concentration applied. Nevertheless, ONOO^−^ has less effect on HuNoV particles.

## Introduction

Human noroviruses (HuNoVs), responsible for acute gastroenteritis, are the leading cause of viral foodborne outbreaks worldwide (Kirk et al., 2015). HuNoVs are a group of genetically diversified viruses belonging to the *Caliciviridae* family with two main genogroups: GI and GII (Chhabra et al., 2019). Genogroup II, genotype 4 (GII.4) HuNoV is the most prevalent in human infections, having caused ~70% of outbreaks since 2002 (van Beek et al., 2018). HuNoVs are coded by a single-stranded, positive-sense RNA genome of ~7,500 nucleotides. The genome is surrounded by an icosahedral capsid. Together, they form virions of 38 nm (Prasad et al., 1999; Jung et al., 2019). The capsid consists of VP1 and VP2 proteins: the major and minor proteins, respectively. VP1 can be subdivided into two domains: the “shell” (S) domain, whose structure determines the geometry of the capsid and the “protruding” (P) domain, which protrudes from the outer part of the capsid and is involved in host-cell interactions (Prasad et al., 1999). The P domain also contains P2 subdomain, which is located on the outermost surface of the capsid (Prasad et al., 1999; Jung et al., 2019). GII.4 HuNoV is structured such that when two P2 subdomains are connected by VP1 dimerization, they form a binding pocket capable of interacting with histo-blood group antigens (HBGAs; Tan et al., 2003, 2004; Cao et al., 2007; Bu et al., 2008), which have been shown to promote HuNoV infection in host cells (Lindesmith et al., 2003; Hutson et al., 2005; Ettayebi et al., 2016).

HBGAs are complex fucose-containing carbohydrates and can be encountered by HuNoVs in the human body in several different contexts. HBGAs are present as free antigens in biological fluids, such as saliva, and are located on red blood cells and mucosal epithelial cells (Huang et al., 2005). The composition of HBGAs varies from individual to individual (Marionneau et al., 2001). This diversity affects their steric hindrances and affinity with the binding pocket of HuNoVs, and thus the human host’s susceptibility to HuNoV infections. The binding pocket of GII.4 HuNoVs is composed of seven highly conserved amino acids, and a peripheral region that can vary in ways that affect its affinity with HBGAs (de Rougemont et al., 2011; Tan and Jiang, 2014). Thus, an individual’s susceptibility to infection is dependent on both HBGA type and HuNoV genotype (Lindesmith et al., 2003; Rockx et al., 2005; Tan and Jiang, 2014; Nordgren and Svensson, 2019).

It is important to define the infectivity of HuNoVs in environmental or food samples. However, this remains difficult because of the lack of cell culture systems that can be used in routine experiments. Stem cell-derived human enteroids and zebrafish larvae have recently been proposed for *in vitro* replication of HuNoVs, but both are still highly complex (Ettayebi et al., 2016; Van Dycke et al., 2019). Any loss of HuNoV infectivity is therefore poorly documented. The molecular mechanisms of viral inactivation are unwell understood, even in culturable enteric viruses (Wigginton et al., 2012; Wigginton and Kohn, 2012; Bastin et al., 2020). In this context, the detection of HuNoVs in foodstuffs and in the environment relies on reverse transcription-quantitative polymerase chain reaction (RT-qPCR). However, such methods cannot be used to determine the infectious risk because viral genome is more stable than viral infectivity (Gassilloud et al., 2003; Polo et al., 2018). Instead of performing direct genome quantification, some authors have suggested first evaluating HuNoV capsid integrity using the HBGA-binding feature to select infectious HuNoVs prior to genome quantification (Moore et al., 2017). To validate this approach, further basic knowledge about the alteration of the capsid protein under inactivating conditions is needed.

Oxidation is one of the gold standard procedures for inactivating enteric viruses including HuNoVs. HuNoVs are transmitted through the fecal-oral route and can therefore be exposed to oxidants during their life cycle. In human hosts, infectious HuNoVs may be inactivated by peroxynitrite (ONOO^−^) and free chlorine (defined as all the chlorine that has not combined with other compounds), which is present in the form of hypochlorous acid (HClO) or hypochlorite ions (ClO^−^), produced by the immune system during inflammation (Masuda et al., 2001; Hawkins et al., 2003; Padalko et al., 2004). Moreover, HuNoVs in the environment can be inactivated by water disinfection treatments, which frequently use free chlorine. Importantly, ONOO^−^ and free chlorine can modify the capsid proteins of HuNoV surrogates and other enteric viruses *in vivo*. However, although oxidants are known to inactivate enteric viruses, the complete molecular mechanisms remain unclear.

In this context, complete GII.4 HuNoV particles from purified human stools and GII.4 virus-like particles (VLPs) were used to evaluate the effects of ONOO^−^ and free chlorine on the HuNoV capsid. The structure of the HuNoV capsid was first investigated using transmission electron microscopy (TEM) and dynamic light scattering (DLS). VP1 integrity was then determined using sodium dodecyl sulfate-polyacrylamide gel electrophoresis (SDS-PAGE). Finally, HBGA-binding to the viral capsid was evaluated in oxidized and non-oxidized particles using enzyme-linked immunosorbent assay (ELISA) for VLPs or RT-qPCR for HuNoVs.

## Materials and Methods

### Production and Purification of GII.4 VLPs

The experiments were performed using GII.4 VLPs (Cairo 4 strain, a 2007 Osaka variant, GenBank: EU876884.1), a GII.4 epidemic variant that have circulated in recent years (Kamel et al., 2009). The GII.4 VLPs were produced using the baculovirus-expressed VP1 system and purified using cesium chloride gradient to a final concentration of 1.36 g.ml^−1^ followed by a centrifugation step at 38,000 rpm for 22 h at 10°C, as previously described (Belliot et al., 2001; de Rougemont et al., 2011). The concentration of GII.4 VLPs was determined using a NanoDrop 2000 spectrophotometer (Nanodrop, Thermo Fisher Scientific, Waltham, MA, United States) at 280 nm (applicable to proteins that contain tryptophan, tyrosine, and phenylalanine) in accordance with the Beer-Lambert equation (i.e., A = Ɛ × l × C when “A” represents the absorbance, “Ɛ” the molar extinction coefficient (l.mol^−1^.cm^−1^), “l” the optical path (cm), and “C” the concentration) and with Ɛ = 57,215 l.mol^−1^.cm^−1^. Purified GII.4 VLPs were diluted to a final concentration of 1 mg.ml^−1^ in TNC buffer (10 mM Tris-HCl, 140 mM NaCl and 10 mM CaCl_2_, pH 7.4) prior to storage at −80°C. The aliquots were thawed extemporaneously as needed prior to each experiment and GII.4 VLPs were diluted in TNC buffer at pH 7.4 to a final concentration of 200 μg.ml^−1^.

### Purification and Concentration of GII.4 HuNoVs

The human feces containing GII.4 HuNoVs were kindly provided by the CHRU of Nancy, France. The genotype of the HuNoVs was confirmed as described previously (Ayouni et al., 2015). GII.4 HuNoVs were purified and concentrated as follows. One gram of fecal suspension of GII.4 HuNoVs [10^9^ genome copies (gc).g^−1^ of stool] was added to 3 ml of 1X PBS solution (137 mM NaCl, 2.7 mM KCl, 10 mM Na_2_HPO_4_, and 1.8 mM KH_2_PO_4_ at pH 7.4). GII.4 HuNoVs were clarified by the addition of 1 ml of chloroform, vortexed for 60 s and centrifuged at 2,500 g for 5 min. The supernatant was submitted to a second clarification step under the same conditions. The recovered samples were then filtered through a 0.22 μm filtration unit (Stericup Millipore Express Plus, Merck Millipore, Merck KGaA, Darmstadt, Germany). Finally, the suspension was purified by ultracentrifugation using a cesium chloride (CsCl) gradient. GII.4 HuNoV suspension (~800 μl) was introduced into a 5-ml tube (Science Services, S9032). A total of 4.2 ml CsCl prepared with 1X PBS to give a final density of 1.44 was then added underneath. The mixture was then ultracentrifuged (OptimaXE-90, rotor SW40 Ti, Beckman Coulter, Brea, California, United States) for 18 h at 160,000 *g* at 4°C. The fraction corresponding to a CsCl density of 1.40 was then collected using a 25G 5/8″ needle. CsCl was then removed by two successive dialyses as previously described (Robin et al., 2019).

Suspensions of GII.4 HuNoVs were stored in the dark, at 4°C, at a final concentration of 3.10^8^ gc.ml^−1^ in 0.1X PBS. The final protein concentration of the GII.4 HuNoV suspensions was estimated at 44 μg.ml^−1^ using a NanoDrop 2000 spectrophotometer (NanoDrop, Thermo Fisher Scientific, Waltham, MA, United States) at 280 nm. The purified and concentrated GII.4 HuNoV suspensions were used within 2 months. The GII.4 HuNoV genome was extracted and quantified using methods described previously (Robin et al., 2019).

### Oxidation of GII.4 VLPs and GII.4 HuNoVs Using Free Chlorine and ONOO^−^

Samples of 20 μl (1 volume) of GII.4 VLP and 1 volume of purified GII.4 HuNoV suspension were subjected to oxidation treatments using free chlorine and ONOO^−^. Oxidations of VLPs and HuNoVs were performed under the same conditions, following previously published protocols (Bastin et al., 2020).

In the free chlorine treatment, 1 volume of GII.4 VLPs or HuNoVs was buffered in 1 volume of 1 M KH_2_PO_4_ at pH 6.5. 1 volume of free chlorine was then added. The reaction was incubated for 10 min at 20°C ± 2°C and the reaction was quenched by the addition of 1 volume of 100 mM sodium thiosulfate. Sodium hypochlorite stock solution (5%, Fisher Scientific, 10573094) was diluted in pure water to obtain our “working solution” of free chlorine at 4 g.l^−1^. This working solution was kept in the dark at 4°C. In each experiment, the free chlorine concentration of the working solution was controlled in accordance with the ISO 7393-2 (2017) procedure using N, N-diethyl-p-phenylenediamine (DPD, Fisher Scientific, 10741325). The final concentrations of free chlorine were 0.1, 0.5, 1, 3, 10, 25, 50, 100, 250, 500, 750, and 1,000 mg.l^−1^ for a contact time of 10 min at 20 ± 2°C. By comparison, the concentration x time (CT) equivalents were 1, 5, 10, 30, 100, 250, 500, 1,000, 2,500, 5,000, 7,500, and 10,000 mg.min.l^−1^. In order to compare the free chlorine treatment with the ONOO^−^ treatment described below, the free chlorine results will be presented in μM [i.e., for the purposes of transposition, we took the molecular weight of HClO (52.46 g.mol^−1^)], giving values of 2, 10, 19, 57, 191, 477, 953, 1,906, 4,766, 9,531, 14,297, and 19,062 μM. No residual free chlorine was detectable after 10 min.

In the ONOO^−^ treatment, 1 volume of GII.4 VLPs or HuNoVs was buffered with 1 volume of 1 M KH_2_PO_4_ at pH 7.4. 1 volume of ONOO^−^ (Interchim, 81565) diluted in 0.3 M NaOH was then added and the reaction occurred at 20 ± 2°C. The final concentrations of ONOO^−^ were 5, 10, 33, 267, and 1,333 μM. The ONOO^−^ reaction is known to occur within seconds at this pH (Goldstein et al., 2005). Therefore, residual ONOO^−^ was not measured after treatment. The ONOO^−^ stock solution was stored at −80°C, and the concentration was measured using spectrophotometric absorption at 302 nm, following the manufacturer’s instructions.

Native and oxidized samples of GII.4 VLPs were then analyzed using TEM, DLS, SDS-PAGE, MALDI-TOF mass spectrometry (MS), and HBGA-binding assays. Native and oxidized samples of GII.4 HuNoVs were analyzed using SDS-PAGE and HBGA-binding assays. TEM, DLS, and MS were not applied to HuNoVs because these approaches are not sensitive enough to be appropriate for use with the purified suspensions used in this study.

### TEM Observations

TEM was used to observe 10 μl of untreated/oxidation-treated GII.4 VLPs following negative staining with phosphotungstic acid, as previously described (Loison et al., 2016). The number of GII.4 VLPs was estimated for each condition using eight representative pictures per field at a magnification of X 15,000. The size of the GII.4 VLPs was quantified using ImageJ software to produce eight representative pictures per field for each condition at a magnification of X 27,500 (Schindelin et al., 2012). This procedure was applied to each oxidant at each concentration in two independent experiments conducted on different days.

### Dynamic Light Scattering

DLS was used to study both the free chlorine/ONOO^−^ treated and the untreated GII.4 VLPs. At the end of the oxidative treatment, 1 ml of 0.1X PBS at pH 7.4 was added, and each sample was filtered through a 0.22 μm cellulose acetate membrane (Stericup Millipore Express Plus, Merck Millipore, Merck KGaA, Darmstadt, Germany) prior to analysis. The hydrodynamic diameter of GII.4 VLPs was measured using the Zetasizer Nano ZS (Malvern Instruments), as described previously (Brié et al., 2016). Diameter measurements were obtained by means of two independent experiments. Triplicate measures were obtained for each sample.

### SDS-PAGE Analysis

SDS-PAGE was used to study both the free chlorine/ONOO^−^ treated and the untreated GII.4 VLPs. They were denatured as previously described, with minor adjustments (Loison et al., 2016). 15 μl of GII.4 VLPs or GII.4 HuNoVs were denatured using 5 μl of 5X Laemmli buffer [2.5 M Tris pH 6.8, 25% (w/v) SDS, 2.5% (w/v) bromophenol blue, 1 M dithiothreitol (DTT), 70% (v/v) glycerol, and distilled water to give a final volume of 10 ml]. Denatured samples were then loaded onto a discontinuous polyacrylamide gel (4% stacking gel and 10% resolving gel) with a standard size marker (Precision Plus Protein Unstained Standards, Biorad, Hercules, CA, United States). Protein migration at 130 V was applied in a Mini-PROTEAN 3 cell set up (Biorad) in a tris-glycine buffer. Gel staining was performed using Oriole fluorescent stain (Biorad) for 60 min and then observed using a Gel Doc system (Loison et al., 2016). The intensity of the bands was measured using ImageJ software (Schindelin et al., 2012). Each protein band was measured across the same surface area, and the intensity of a protein band was determined as follows: maximum background light intensity – mean light intensity. The intensity of each protein band was defined for untreated samples (I_0_) and oxidized samples (I). The ratio I/I_0_ was used to control the evolution of intensities. The intensities were obtained by means of three independent experiments conducted on different days.

### MALDI-TOF MS

Following the separation of GII.4 VLP proteins using SDS-PAGE, the protein bands were excised under a UV lamp at 253 nm using a scalpel and analyzed using MALDI-TOF MS to confirm the identity of the VP1 protein. The protein extraction method was inspired by that described by Gundry et al. (2009) with modifications. Each band of interest was excised and diced into ~1 mm^3^ pieces. The pieces of diced gel were destained using 150 μl of solution (i.e., 50% acetonitrile (ACN) and 50% 50 mM NH_4_CO_3_ at pH 8.0) and incubated at room temperature for 30 min. For the purposes of dehydration, the solution was then replaced with 100% ACN at room temperature for 10 min. To reduce disulfide bonds and alkylate free cysteines, the gel pieces were treated with a 10 mM DTT solution at 37°C for 30 min, followed by a further 30 min of 150 mM iodoacetamide solution in the dark at room temperature. Two more destaining treatments were applied for 15 min each, followed by a dehydration treatment involving incubation at room temperature for 10 min. The supernatant was then removed and the pieces of gel were air-dried at room temperature. Fifty microliter of 50 mM NH_4_CO_3_ pH 8.0 containing 1 μg of trypsin (T6567 Merck) were then added and the solution was incubated on ice for 1 h. Excess trypsin was removed and replaced by a large enough volume of 50 mM NH_4_CO_3_ pH 8.0 to cover the pieces of gel. The samples were then kept at 37°C for 18 h. Tryptic peptides were extracted by vortexing for ~1 min and centrifuging at 12 rpm for 5 min. The solution was then transferred to a new tube and stored at 4°C prior to analysis using MALDI-TOF MS. The matrices and MALDI-TOF MS methods were the ones previously used (Bastin et al., 2020).

Specific capsid protein peptide mass fingerprints were determined using MALDI-TOF MS. Theoretical masses were obtained by theoretical digestion of VP1 capsid proteins (GII.4.VLP proteins) using the online software, PeptideMass from ExPaSy.^1^ Acrylamide adduct and up to one missed cleavage were taken into account in this analysis. A peptide mass accuracy of up to 20 ppm was tolerated. The VP1 sequence used for reference had the following accession numbers: GenBank: EU876884.1. Masses that were found in the blank gel (SDS-PAGE gel stained with Oriole, signaled free from proteins following the same extraction and trypsin digestion protocol) and across protein samples were disregarded, as such masses would be autolyze or trypsin peptides or human contaminants. Two independent experiments were performed.

### Saliva Collection and HBGA Extraction

Saliva samples from 15 healthy adult volunteers were collected using the following procedures and approved by the Nantes University Hospital Review Board (study number BRD02/2-P; de Rougemont et al., 2011). Consent was obtained from all donors, and all experiments were performed in accordance with the relevant guidelines and regulations. After collection, the human saliva samples were boiled at 100°C for 5 min and then centrifuged at 10,000 g for 5 min. Supernatants containing HBGAs were stored at −20°C until needed. ELISA was used to determine the presence of A, B, and O blood group antigens and Lewis antigens in all samples, as described previously (Huang et al., 2005).

### HBGA-Binding Assays

HBGA-binding assays were performed on GII.4 VLPs and GII.4 HuNoVs treated or untreated with free chlorine or ONOO^−^ using the previously described methods (Robin et al., 2019; Chassaing et al., 2020). In both the VLP and HuNoV HBGA-binding experiments, human saliva treated with sodium periodate was systematically introduced as a negative control. One hundred microliter of native or free chlorine/ONOO^−^ treated GII.4 VLPs, diluted to 0.1, 0.5, and 1.0 μg.ml^−1^ were analyzed. HBGA-binding assays were carried out using ELISA (Robin et al., 2019). In the case of HuNoVs, 100 μl of native and oxidized suspensions pre-treated with RNase A (as described hereafter) were analyzed. HBGA-binding to the viral capsid was then quantified using RNA amplification by RT-qPCR, following the extraction and purification of the viral genome. Lewis-positive (ALe^+^) saliva samples were used for GII.4 VLPs and HuNoVs because they had the highest relative affinity of the human saliva tested under our experimental conditions. Results were obtained by means of three independent experiments. Duplicate measures were taken for each sample.

### RNase Treatment

All GII.4 HuNoV samples were submitted to RNase treatment in order to eliminate capsid-free RNA genome, as described previously (Brié et al., 2016). To validate the efficacy of the RNase, 20 μl of 10^6^ gc.ml^−1^ of capsid-free MS2 phage RNA genome was added to each sample as an internal control. RNase A (Thermo Fischer Scientific, EN0531) was added to reach a final concentration of 100 μg.ml^−1^. The reaction was incubated for 1 h at 37°C. The MS2 phage and GII.4 HuNoV genomes were then extracted and quantified using the appropriate RT-qPCR primers, probes, and procedures (Ogorzaly and Gantzer, 2007; ISO 15216-1, 2017). We waited until RT-qPCR failed to detect any MS2 phage genome, in order to ensure that we were only quantifying encapsidated HuNoV RNA genome.

### Statistical Analysis

All statistical analyses were performed using R statistical software (Rx64 v.3.5.3; R Core Team, 2019). A Shapiro-Wilk test with a chosen alpha level of 0.05 was performed to confirm the normality of the data. A paired or unpaired sample Student’s t-test was performed on dependent and independent data that followed a normal distribution, respectively. Dependent and independent data with a non-normal distribution were subjected to a Wilcoxon signed-rank test or a Mann–Whitney U test. The significance level of all tests was set to 0.05.

## Results

### Effects of Free Chlorine and ONOO^−^ on GII.4 VLP Structure

With the GII.4 VLPs, modifications of capsid structure were evaluated using two criteria: size and number of particles. Three concentrations were tested for each oxidant (953; 4,766 and 9,531 μM for free chlorine for 10 min and 33; 267 and 1,333 μM for ONOO^−^) at 20 ± 2°C. DLS allows the hydrodynamic diameter of the particles to be measured. The mean size of native GII.4 VLPs in the control suspension was 49.9 ± 3.2 nm (Table 1). Treatments using free chlorine and ONOO^−^ slightly increased the size of VLPs in suspension. The size increased significantly to 68.3 and 79.8 nm when the concentrations of free chlorine and ONOO^−^ reached 9,531 and 1,333 μM, respectively (i.e., *p* < 0.01). The polydispersity index (PdI), which can define the size distribution (i.e., monodisperse for values below 0.1 and polydisperse for values above 0.1) was not significantly modified by the treatments. The mean value was about 0.258 (Table 1). This PdI value indicates a polydisperse VLP suspension (i.e., a suspension with a range of particle sizes). Nevertheless, quality control of size measurement was also validated as PdI was less than 0.7 (Malvern Instruments Limited, 2011). Another quality control value is based on Y intercept (i.e., signal-to-noise ratio) which should be over 0.6 (Malvern Instruments Limited, 2011). This value was about 0.76 ± 0.04 for the control, and gradually decreased with increases in the concentration of oxidative treatments (Table 1). At the critical concentrations of 9,531 μM of free chlorine and 1,333 μM of ONOO^−^, the Y intercept was below 0.6, indicating a significant decrease in the quantity of VLPs. The decrease may be due to the dissociation of VLPs/aggregation of VP1 proteins. Part of VP1 aggregates may stay on the 0.22 μm used before DLS measurements.

**Table 1:** Size of GII.4 VLPs after oxidative treatments defined by DLS.

	Control	Free chlorine (μM)			ONOO^−^ (μM)		
		953	4,766	9,531	33	267	1,333
Size (nm)	49.9 ± 3.2	53.7 ± 2.3	56.1 ± 2.4	68.3^*^ ± 1.4	64.9 ± 4.6	72.9 ± 9.4	79.8^*^ ± 3.4
PdI^$^	0.297 ± 0.08	0.297 ± 0.05	0.194 ± 0.02	0.200 ± 0.05	0.301 ± 0.02	0.255 ± 0.02	0.261 ± 0.03
Y^$$^	0.76 ± 0.04	0.74 ± 0.05	0.70 ± 0.01	0.53 ± 0.02	0.71 ± 0.04	0.64 ± 0.01	0.44 ± 0.04

* Unpaired student’s *t*-test, *p* < 0.01.

$ Polydispersity index.

$$ Intercept value.

**Figure 1:**
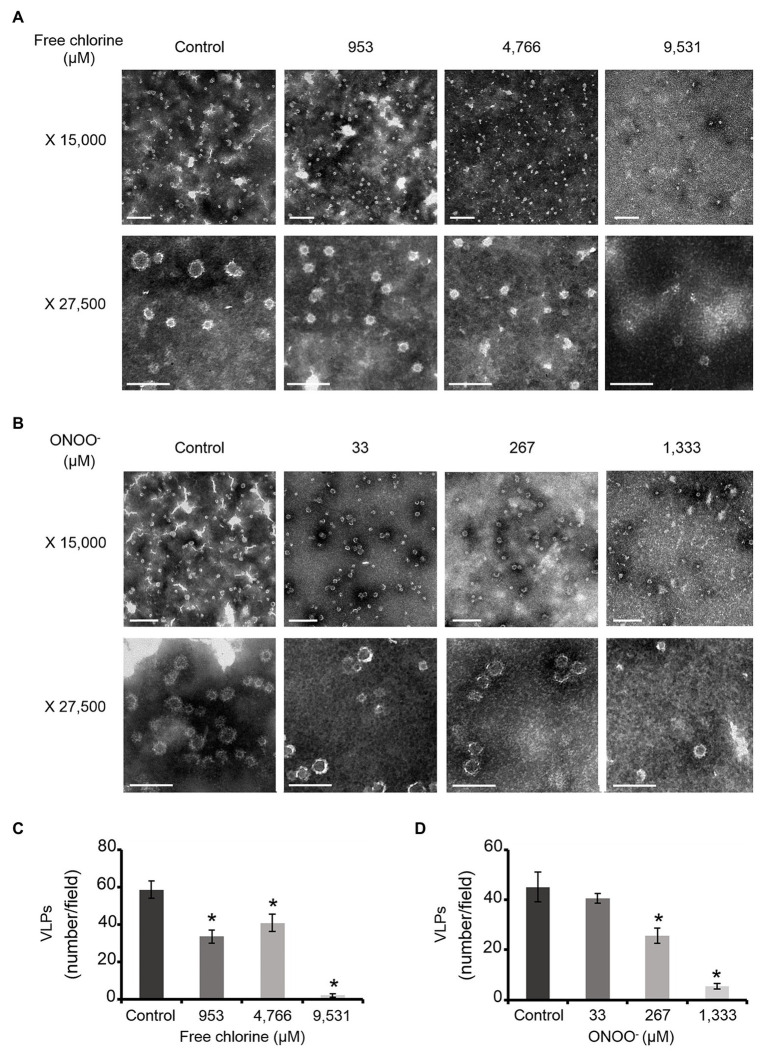
Morphology of GII.4 VLPs after oxidative treatments. Images of GII.4 VLPs, both untreated and treated with free chlorine **(A)** and ONOO^−^ **(B)** captured using TEM at X 15,000 (white scale bar at 0.2 μm) and X 27,500 (white scale bar at 100 nm). Representative numbers of GII.4 VLPs per field observed using TEM at X 15,000 are indicated for native and oxidized particles at different concentrations of free chlorine **(C)** and ONOO^−^ **(D)**. The number of GII.4 VLPs was determined from eight representative fields, which were obtained from two independent experiments, conducted on different days. Error bars indicate standard deviations. *Unpaired Student’s *t*-test and Mann-Whitney U test were used to compare groups with *p* < 0.01.

TEM observations enabled us to determine particle diameters and to estimate the numbers of VLPs (Figure 1). Two types of particles were identified in untreated VLPs at a magnification of X 27,500: one population centered around 23 ± 3 nm and another around 34 ± 6 nm (Figures 1A,B). The smaller particles represented around 60% of total VLPs. At a magnification of X 15,000, some 45–60 VLPs were observed per field (Figure 1). Treatments with both oxidants slightly decreased the number of VLPs in intermediate concentrations, in which around 20–40 particles per field were counted (Figure 1). The critical concentrations of 9,531 μM of free chlorine and 1,333 μM of ONOO^−^ led to a substantial decrease in the number of VLPs (to below 5 VLPs per field; Figure 1). However, the reduction of the number of VLPs estimated by TEM can be slightly influenced by the deposition properties of the different VLPs on the TEM grid depending to the study design and the disinfectant used.

Together, DLS and TEM analyses showed that GII.4 VLPs are polydispersed in suspension, with smaller and larger particles. The critical concentrations of 9,531 μM of free chlorine and 1,333 μM of ONOO^−^ led to significant disruption of the VLPs. At lower concentrations of both oxidants, there was no obvious modification to particle size or number of VLPs.

### Effects of Free Chlorine and ONOO^−^ on the Major Capsid Protein (VP1) of GII.4 VLPs

**Figure 2:**
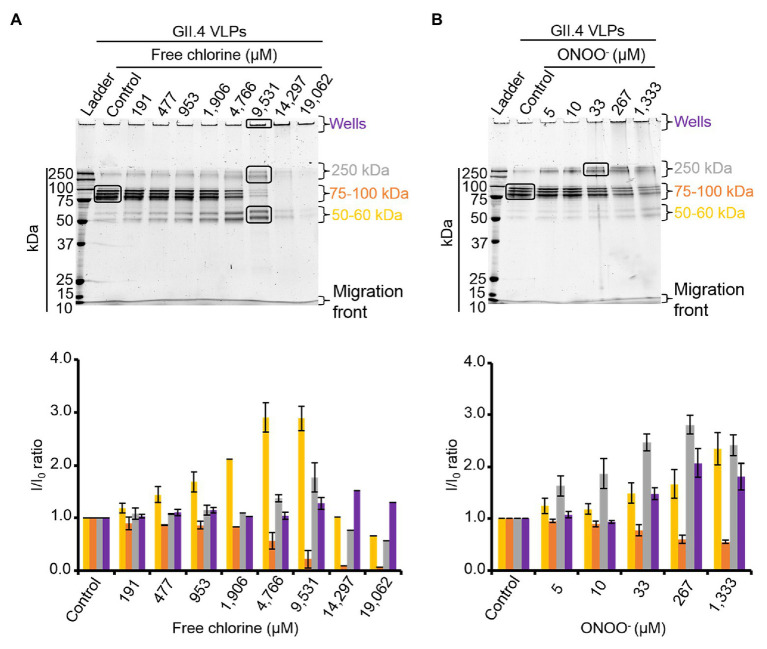
Free chlorine and ONOO^−^ modify the capsid proteins of GII.4 VLPs. GII.4 VLPs were analyzed using SDS-PAGE following treatment with free chlorine **(A)** or ONOO^−^ **(B)** without heat treatment. The variation in intensity of protein bands obtained using SDS-PAGE gel was measured for both oxidative treatments (see the histograms placed below the gels of the respective conditions). The intensity of protein bands (I/I_0_ ratio) was determined by dividing the mean intensity value of each band obtained at a specific molecular weight following oxidative treatment (I) by the mean intensity value of the same band obtained in the untreated control (I_0_). Each data point is an average of three independent experiments conducted on different days (except for VLPs treated with free chlorine at final concentrations of 14,297 and 19,062 μM, for which one replication was performed). The intensity of protein bands was separated into groups, as indicated by the color code on the right-hand side of the gels (50–60 kDa, 75–100 kDa, and 250 kDa, and the wells are in yellow, orange, gray, and violet, respectively). Error bars indicate standard deviations. Black rectangles on SDS-PAGE gels correspond to the protein bands that were analyzed using MS.

The effects of free chlorine and ONOO^−^ on the VP1 of GII.4 VLPs were evaluated using SDS-PAGE. DTT and SDS were used to produce denaturing conditions to promote the dissociation of VLPs into VP1 monomers. Notably, native VP1 monomers appeared at their expected masses of 57 and 60 kDa (de Rougemont et al., 2011; Huo et al., 2015; Figures 2A,B). Both VP1 masses have been previously explained by the presence or absence of an N-terminal amino acid sequence (Huo et al., 2015). Three main bands were observed between 75 and 100 kDa (Figures 2A,B). MALDI-TOF MS confirmed that they contained VP1 (Supplementary Figure S1). They may correspond to different kinds of VP1 dimers, only some of which contain the N-terminal sequence (Someya et al., 2011). Finally, a single weak band at ~250 kDa was also identified as VP1 by MALDI-TOF MS (Figure 2; Supplementary Figure S1). This probably corresponds to VP1 aggregate forms. Heating at 95°C for 10 min prior to SDS-PAGE analysis confirmed the presence of VP1 in those aggregates (Supplementary Figure S2). Notably, after heating at 95°C, only the two expected masses of VP1 at 57 and 60 kDa were observed and the intensity of the protein bands had increased by comparison with the unheated samples (Supplementary Figure S2). Together, these data demonstrated that SDS-PAGE analysis under denaturing conditions, without a heating at 95°C, was not sufficient to completely dissociate VLPs into VP1 monomers. VP1 dimers were mainly observed and some VP1 aggregates were also still present (Figures 2A,B).

As previously observed in HuNoV surrogates, free chlorine and ONOO^−^ treatments changed the SDS-PAGE profiles of VP1 under denaturing conditions (Figures 2A,B; Bastin et al., 2020). The intensity of the VP1 dimers at ~75–100 kDa decreased in a dose-response manner until the critical free chlorine concentration of 9,531 μM had been reached (Figure 2A). Simultaneously, VP1 monomers at ~50–60 kDa increased significantly, and VP1 aggregates at ~250 kDa increased moderately (Figure 2A). This result suggests that free chlorine affects the distribution of VP1 monomers, dimers, and aggregates under our experimental conditions. Following treatment with free chlorine at above 9,531 μM, the intensity of all protein bands from 10–250 kDa decreased, without the formation of new bands (Figure 2A). Cleavage into smaller peptides with variable masses or high aggregation – implying that aggregates remained in the starting well – may therefore have occurred upon treatment with free chlorine.

The SDS-PAGE protein migration profiles of VLPs treated with ONOO^−^ displayed similar changes to those of VLPs treated with free chlorine, though there were some differences. Notably, the intensity of VP1 aggregates at ~250 kDa increased in a dose-dependent manner until the ONOO^−^ concentration reached 1,333 μM (Figure 2B). VP1 monomers at ~50–60 kDa also increased, though to a lesser extent. Conversely, the intensity of VP1 dimers at ~75–100 kDa decreased with increasing ONOO^−^ concentrations (Figure 2B). ONOO^−^ treatment resulted in the formation of more VP1 aggregates than VP1 monomers and in the formation of more VP1 aggregates than free chlorine treatment (Figures 2A,B).

Together, these data show that free chlorine and ONOO^−^ treatments change the propensity of GII.4 VLPs to dissociate under SDS-PAGE denaturing conditions. With free chlorine at higher concentrations than 9,531 μM, the overall VP1 protein signal decreases in all areas between 10 and 250 kDa, suggesting more profound modifications to VP1.

### Effects of Free Chlorine and ONOO^−^ on HBGA-Binding to GII.4 VLPs

**Figure 3:**
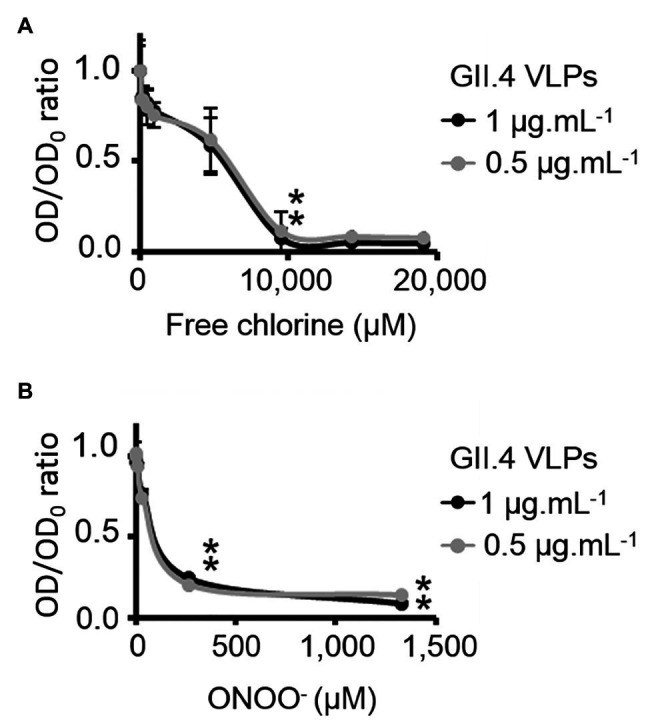
Free chlorine and ONOO^−^ prevent HBGA-binding to GII.4 VLPs. Assays of HBGA-binding to GII.4 VLPs using ALe^+^ saliva following free chlorine **(A)** and ONOO^−^ **(B)** treatments were presented. The OD/OD_0_ ratio values were determined by dividing the mean OD_450_ values obtained by HBGA-binding ELISA at each oxidant concentration (OD) by the mean OD_450_ values obtained in the untreated control (OD_0_). Two concentrations of GII.4 VLPs were tested: 1 μg.ml^−1^ (black circles and line) and 0.5 μg.ml^−1^ (gray circles and line). Each data point corresponds to the mean OD_450_ values emanating from three independent experiments with duplicate measures carried out on different days (except for VLPs treated with free chlorine at final concentrations of 14,297 and 19,062 μM, for which one replication was performed). Error bars indicate standard deviations. *Unpaired Student’s *t*-test was used to compare groups with *p* < 0.01.

HBGA-binding to GII.4 VLPs was evaluated using ELISA. The specific interactions between ALe^+^ and VLPs were confirmed using sodium periodate, which cleaves these complex carbohydrates and thus preventing the specific binding to the capsid (Maalouf et al., 2010; Supplementary Figure S3). Using 2- and 10-fold dilutions of VLPs, a progressive reduction in HBGA-binding to VLPs was observed (Supplementary Figure S3). Notably, this set up allowed for the linear reduction of absorbance, producing an up to 90% reduction in HBGA binding to VLPs (Supplementary Figure S3). In view of this, only VLPs at 1.0 and 0.5 μg.ml^−1^ were used in order to stay within this dynamic range. In GII.4 VLPs, only VP1 dimers are able to interact with HBGAs (Tan et al., 2003, 2004; Cao et al., 2007; Bu et al., 2008). The results obtained using SDS-PAGE suggest that oxidation produces changes in the affinity of VP1 dimers (Figure 2) and indicate that HBGA-binding to VLPs could decrease accordingly. Indeed, there was evidence of a progressive reduction in VLP binding to ALe^+^ saliva with increasing concentrations of free chlorine or ONOO^−^ (Figure 3). Moreover, free chlorine at 9,531 μM clearly induced more than 90% reduction in HBGA-binding to VLPs, since the OD/OD_0_ ratio was below the limit of detection (Figure 3A). Interestingly, this same concentration of free chlorine induced major changes in the SDS-PAGE profiles of VP1 under denaturing conditions (Figure 2A), as well as a significant reduction in the number of VLPs per field, observed using TEM (Figure 1A). ONOO^−^ at 267 and 1,333 μM also significantly decreased the amount of HBGA-binding to VLPs. At 1,333 μM of ONOO^−^, more than 90% of VLPs were not able to bind to ALe^+^ saliva (Figure 3B). Important changes to the SDS-PAGE profiles of VP1 under denaturing conditions and a significant reduction in the number of VLPs with the same concentration of ONOO^−^ were also observed, using TEM (Figures 1B, 2B).

Overall, these data showed that GII.4 VLPs lose their capacity to bind to HBGAs at a free chlorine concentration of 9,531 μM, which produces loss of VLP structure. The same reduction in HBGA-binding was observed in VLPs treated with 267 μM of ONOO^−^, though TEM and DLS determined that ONOO^−^ at this concentration did not produce loss of capsid structure.

### Effects of Free Chlorine and ONOO^−^ on the Capsid Proteins of GII.4 HuNoVs

**Figure 4:**
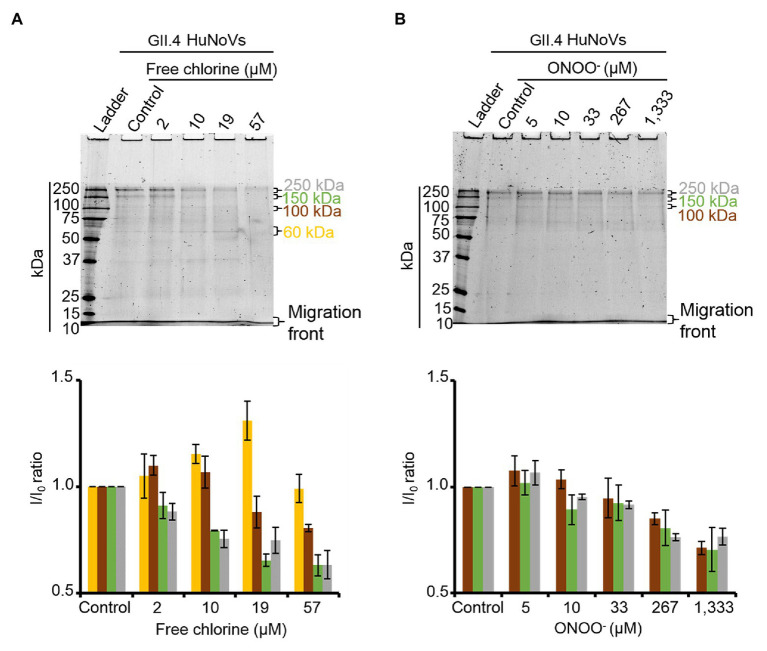
Free chlorine and ONOO^−^ modify the capsid proteins of GII.4 HuNoVs. GII.4 HuNoVs were analyzed using SDS-PAGE following free chlorine **(A)** or ONOO^−^ **(B)** treatments without heat treatment. The variations in intensity of protein bands obtained using SDS-PAGE gel were measured for both oxidative treatments (see histograms below the gels of the respective conditions). The intensity of protein bands (I/I_0_ ratio) was determined by dividing the mean intensity value of each band obtained at a specific molecular weight after oxidative treatment (I) by the mean intensity value of the same bands obtained in the control without oxidant (I_0_). Each data point is an average of three independent experiments conducted on different days. The intensity of the protein bands was separated into groups, as indicated by the color code on the right-hand side of the gels (60 kDa, 100 kDa, 150 kDa, and 250 kDa are shown in yellow, brown, green, and gray colors, respectively). Error bars indicate standard deviations.

To validate the results obtained with GII.4 VLPs, SDS-PAGE analysis was performed on GII.4 HuNoVs before and after oxidative treatments. Under denaturing conditions, purified GII.4 HuNoVs from human stools displayed a different SDS-PAGE profile from that of GII.4 VLPs following free chlorine treatments performed under the same conditions (Supplementary Figure S4). Only for this experiment, the VLP protein were diluted 10 times to have similar concentrations of proteins compared to HuNoVs (i.e., 20 μg.ml^−1^ for VLPs and 44 μg.ml^−1^ for HuNoVs.) in the SDS-PAGE profile. In GII.4 HuNoVs, protein bands could only be observed at ~100, 150, and 250 kDa. Moreover, although the concentrations of ONOO^−^ used to treat GII.4 HuNoVs were comparable to those used to treat GII.4 VLPs, the concentrations of free chlorine were decreased in order to allow SDS-PAGE to continue to detect protein bands. When the same free chlorine concentrations used to treat VLPs were applied to HuNoVs, SDS-PAGE was unable to observe any protein bands at all (data not shown). This could be explained by the initial protein concentration in VLPs, which was approximately 5-fold higher (200 μg.ml^−1^) than those in HuNoVs (44 μg.ml^−1^) for the majority of the experiments and this is why we tried also with 10 times diluted VLPs (Supplementary Figure S4). The results being exactly the same for VLPs this might not be the only explanation. For HuNoV, the free chlorine concentrations tested therefore ranged from 2 to 57 μM. These treatments progressively decreased the intensity of the most representative protein bands (~100, 150, and 250 kDa) and increased the intensity of a protein band equivalent to VP1 monomers at ~60 kDa (Figure 4A). With free chlorine at 57 μM, the intensity of all the GII.4 HuNoV protein bands decreased compared to those of untreated HuNoVs (Figure 4A). This data suggest that the GII.4 HuNoV capsid proteins were (i) more sensitive to free chlorine treatment than GII.4 VLPs, (ii) were modified in regions involved in dimer stability, and (iii) were highly disrupted at above a critical concentration of 19 μM. ONOO^−^ treatments of 5–1,333 μM induced more moderate changes to the SDS-PAGE profiles of GII.4 HuNoVs (Figure 4B). Notably, 0–33 μM ONOO^−^ did not change the relative intensity of each protein band at ~250, ~150, and ~100 kDa. ONOO^−^ treatments of 267 and 1,333 μM decreased the intensities of all protein bands by 20 and 30%, respectively, compared to the untreated condition. Finally, no band equivalent to VP1 monomers was observed.

### Effects of Free Chlorine and ONOO^−^ on HBGA-Binding to GII.4 HuNoVs

**Figure 5:**
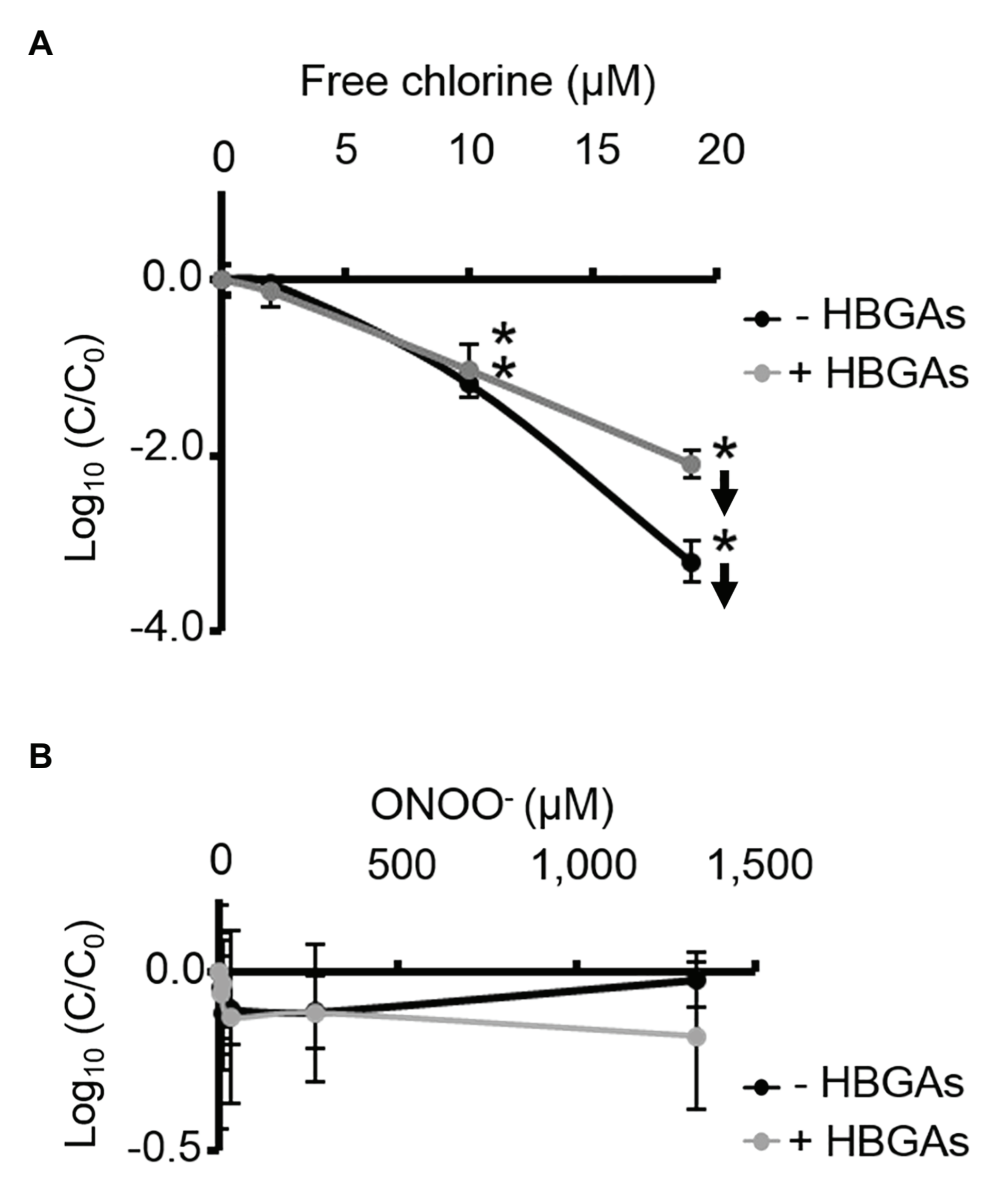
Free chlorine and ONOO^−^ decrease HBGA-binding to complete GII.4 HuNoVs. Assays of HBGA-binding to encapsidated GII.4 HuNoVs in ALe^+^ saliva following free chlorine **(A)** and ONOO^−^ **(B)** treatments were presented. Complete HuNoVs correspond to viral particles treated with RNase at a final concentration of 100 μg.ml^−1^ for 1 h at 37°C. The black line and gray line represent complete HuNoVs quantified by RT-qPCR and complete HuNoVs after HBGA-binding followed by RNA amplification using RT-qPCR, respectively. The log (C/C_0_) values were calculated by dividing the mean GII.4 HuNoV gc values following oxidative treatment (C) by the mean GII.4 HuNoV gc values in the untreated control (C_0_). Each data point corresponds to the mean data value of duplicates of three independent experiments carried out on different days. Error bars indicate standard deviations. Arrows (↓) indicate data below the quantification limit. *Unpaired Student’s *t*-test and Mann-Whitney U tests were used to compare groups with *p* < 0.01.

ALe^+^ saliva was used to evaluate changes in the binding capacity of GII.4 HuNoVs to HBGAs following oxidative treatments. Interestingly, free chlorine treatment produced significant dose-dependent decreases in the ability of HuNoVs to interact with HBGAs (Figure 5A). Free chlorine treatments of 10–19 μM decreased HBGA-binding to HuNoVs by more than 90–99% (i.e., 1.0–2.1 log_10_; Figure 5A). Importantly, 19 μM of free chlorine induced a major decrease in the GII.4 HuNoV capsid protein signals observed using SDS-PAGE (Figure 4A).

Direct quantification of the viral genome showed that log_10_ reductions in HuNoVs were similar to those observed following HBGA-binding assays prior to genome quantification following treatment with 19 μM of free chlorine. ONOO^−^ treatments did not reduce HBGA-binding to GII.4 HuNoVs – even at 1,333 μM (Figure 5B), a concentration able to dissociate GII.4 VLP capsid proteins (Figure 1B). There was a consistent failure to observe any reduction in HuNoVs following direct genome quantification over the whole range of ONOO^−^ treatments.

Free chlorine and ONOO^−^ treatments both produce observable differences in GII.4 HuNoV phenotypes. The former oxidative treatment may prevent interaction with HBGAs because of the dissociation of capsid proteins, especially VP1 dimers, once the critical concentration of 19 μM has been reached. This critical concentration is much lower for GII.4 HuNoVs than for GII.4 VLPs under the same conditions. The latter oxidative treatment has no clear effect on GII.4 HuNoVs in terms of SDS-PAGE capsid protein migration or HBGA-binding to capsids, even at a concentration that induces the dissociation of VLP capsid proteins.

## Discussion

This study was undertaken to define the mechanisms that alter the HuNoV capsids after free chlorine and ONOO^−^ treatments. Both GII.4 VLPs and HuNoVs were investigated using different and complementary approaches.

The purified GII.4 VLPs used in our study were established to be between 23 ± 3 and 34 ± 6 nm in size, using TEM. This particle size heterogeneity was explained by the absence or presence of the N-terminal sequence in VP1, as described previously (Huo et al., 2015). However, both kinds of particles express similar antigenic properties (White et al., 1997). The smaller and larger VLPs represent T = 1 (60 VP1 subunits) and T = 3 (180 VP1 subunits) icosahedral symmetry, respectively (White et al., 1997). Complete GII.4 HuNoVs, corresponding to viral particles from purified human stools, treated using RNase, were also studied. HuNoVs definitely exhibit a T = 3 symmetry including VP1 and VP2. This allows us to mention that there is a report that there may be a genotype-dependent variation in the organization of the capsid that has been observed with VLPs (Jung et al., 2019).

SDS-PAGE analysis of VLPs under denaturing conditions demonstrated that VP1 dimers were the major form of VLPs, together with, to a lesser extent, monomers and aggregates, as has previously been observed under such conditions (Someya et al., 2011). VP1 dimers are highly stable even under denaturing conditions such as urea (Tan et al., 2004). It has been observed that the P domain of VP1 is essential to dimer formation and only the VP1 dimers of GII.4 VLPs were able to interact with HBGAs since residues of the P domain which interact with these ligands are found on both monomers (Cao et al., 2007; Bu et al., 2008). VP1 aggregates, such as those observed on SDS-PAGE at ~250 kDa, have previously been described as possible intermediates in the morphogenesis of VLPs (Tresset et al., 2013). In complete HuNoVs under the same conditions, only VP1 aggregates were observed, which suggests that HuNoV capsids are more stable than VLPs.

Our study refined our understanding of the effects of free chlorine treatment on GII.4 HuNoV capsids. In France, the usual CT values for free chlorine applied in disinfection processes are around 10 and 40 mg.min.l^−1^ for water and food, respectively (Legifrance, 2006; Boudaud et al., 2012). The CT values required to reach 1.0–2.0 log_10_ reduction in viral inactivation range from 0.1 to 300 mg.min.l^−1^ for HuNoV surrogates such as canine calicivirus (CaCV), murine norovirus (MNV), and hepatitis A virus (HAV; Abad et al., 1994; Li et al., 2002; Duizer et al., 2004; de Abreu Corrêa et al., 2012; Dunkin et al., 2017). Brié et al. (2017) produced a 4.0 log_10_ reduction in MNV using a CT value of 750 mg.min.l^−1^. The CT values applied to HuNoVs in our study ranged from 1.0 to 10,000 mg.min.l^−1^ and therefore encompassed the entire spectrum of values used worldwide. Our results demonstrated that different mechanisms govern the degradation of HuNoV capsids after free chlorine treatments, depending on the CT value used.

The first step was the modification of amino acid residues directly or indirectly involved in VP1 dimer stabilization without significant disruption of VLPs. This was observed to occur from the initial free chlorine concentration of 191 μM (CT value of 100 mg.min.l^−1^) until a critical concentration of 9,531 μM (CT value of 5,000 mg.min.l^−1^) with a dose-dependent increase in particle size. Previous studies suggest that both hydrogen bonds and hydrophobic interactions are important in stabilizing VP1 dimers (Cao et al., 2007; Bu et al., 2008). In GII.4 HuNoV strain (VA387), 57 residues have been observed of each VP1 subunit with a solvent-accessible surface that is buried upon dimerization (Cao et al., 2007), including methionine, cysteine, histidine, tryptophan, tyrosine, and lysine amino acid residues, which are known to be highly sensitive to free chlorine, such as we observed higher oxidation of M237 and M333 by MS (not shown). Nevertheless, it is possible that the oxidants induced N373 deamidation, which may also be a factor to consider for the stability of the particle and thus adhesion to HBGAs (Mallagaray et al., 2019).

At higher concentrations of free chlorine of 4,766 μM (CT value of 2,500 mg.min.l^−1^), cross-links may have formed between VP1 monomers, increasing the presence of VP1 aggregates detected by SDS-PAGE at 250 kDa. The formation of cross-links between VP1 monomers has been described previously (Meunier et al., 2004; Bastin et al., 2020) and is involved in the inactivation mechanisms of coxsackievirus (Padalko et al., 2004), another pathogenic enteric virus that infects humans.

At the critical concentration of 9,531 μM free chlorine (CT value of 5,000 mg.min.l^−1^), VLPs were completely disrupted, as demonstrated by TEM and SDS-PAGE analysis. This CT value is higher than the 500 mg.min.l^−1^ used in a previous study that observed the same effects (Sato et al., 2016). However, the same authors were working on a different VLP genotype (GI.4; Sato et al., 2016). In our study, a free chlorine concentration of 9,531 μM led to the disruption of VLPs and to a significant reduction in HBGA-binding. Lower concentrations of free chlorine either did not affect the binding pocket at all or not enough to prevent HBGA-binding. Given that we know that VP1 dimers are required in interactions with HBGAs, the alteration of the dimers readily explains the reduction in HBGA-binding to viral capsids. Moreover, a free chlorine concentration of above 9,531 μM significantly altered VP1, leading to a complete loss of protein signal. Sato et al. (2016) also achieved consistent results using SDS-PAGE, demonstrating that the number of VP1 monomers first increases and then decreases with increasing concentrations of free chlorine.

The concentration of free chlorine used on complete HuNoVs was drastically reduced from that used on VLPs in order to retain visible protein bands under SDS-PAGE analysis. Our results suggested that complete HuNoVs could be more sensitive to free chlorine treatment, compared to VLPs. Whereas concentrations of 191–9,531 μM (CT values of 100–5,000 mg.min.l^−1^) were applied to VLPs, concentrations of 2–57 μM (CT values of 1–30 mg.min.l^−1^) were more appropriate for complete HuNoVs. This difference can be readily explained by the higher initial protein concentration in VLPs (200 μg.ml^−1^) compared to HuNoVs (44 μg.ml^−1^) but not because the decrease in VLPs by factor 10 give exactly the same results. The choice to use a higher concentration of VLPs was motivated by the high protein content required by the DLS, TEM, and MS approaches. Our results showed modifications to purified and complete HuNoV capsids over the entire range of CT values. As described previously, CT values ranging from 1 to 5 mg.min.l^−1^ led to a 1.0–2.0 log_10_ reduction in the genomes of purified HuNoVs (Shin and Sobsey, 2008; Kitajima et al., 2010; Sano et al., 2010). Conversely, a 0.4 log_10_ reduction in genomes was observed in non-purified HuNoVs, using CT values of 2,500 mg.min.l^−1^ (Tung et al., 2013). Studies of feline calicivirus (FCV) also found that the more viruses were purified, the more sensitive to free chlorine they became (Urakami et al., 2007). Thus, the matrix effect obviously plays an important role in virus inactivation during oxidative treatment. In addition to the simple removal of the protective matrix, it is possible that the purification steps of HuNoVs with CsCl could potentially make the particles somewhat more sensitive to oxidative treatment (Huhti et al., 2010). In both GII.4 VLPs and complete GII.4 HuNoVs, an increase in VP1 monomers was observed with the increase in free chlorine, which once again suggests a loss of VP1 self-affinity. In addition, the instability of the VP1 dimers was correlated with the loss of HBGA-binding to the binding pocket. Wang and Tian, (2014) showed a 2.3 log_10_ reduction in HBGA-binding to GII.4 HuNoVs using CT values of 80 mg.min.l^−1^. Kingsley et al. (2014) demonstrated 1.5 and 4.0 log_10_ reductions in HBGA-binding to GI.1 HuNoVs at CT values of 33 and 173 mg.min.l^−1^, respectively. Given our results, it is reasonable to suggest that the VP1 dimers of GII.4 and GI.1 HuNoVs may be disrupted at the concentrations used by these authors.

Our study also refined our understanding of the effects of ONOO^−^ treatment on GII.4 HuNoV capsids. As the ONOO^−^ reaction is known to be very fast, CT values were not calculated (Goldstein et al., 2005). The oxidant is physiologically produced in humans and can also be produced by a dielectric barrier discharge (DBD) plasma torch (Yamashiro et al., 2018). At concentrations of 2,000 and 800 μM, ONOO^−^ has been found to inactivate 1.0–2 log_10_ of virus surrogates of FCV and Qβ or MS2 phages, respectively (Yamashiro et al., 2018; Bastin et al., 2020). Different stages of the degradation of HuNoV capsids were observed after ONOO^−^ treatment. At lower concentrations of 5–267 μM, an increase in VP1 aggregates can be observed using SDS-PAGE under denaturing conditions. This could be induced by changes to the hydrophobic interactions of monomers or by oxidation-induced cross-linking (Padalko et al., 2004; Bastin et al., 2020). The dissociation of VLPs was not observed in this concentration range. At the same time, significant decreases in HBGA-binding to viral capsids were observed from 267 μM. This suggests direct or indirect changes to the binding pocket. ONOO^−^ not only preferentially oxidizes cysteine and methionine but also affects tyrosine and tryptophan (Alvarez and Radi, 2003; Hawkins et al., 2003). Interestingly, the GII.4 HuNoV binding pocket is composed of seven amino acids (Ser343, Thr344, Arg345, Asp374, Ser441, Gly442, and Tyr443) which are conserved in the GII.4 genotypes (Tan and Jiang, 2014). At the critical ONOO^−^ concentration of 1,333 μM, the VLPs were dissociated. The results obtained with VLPs were not corroborated with complete HuNoVs, which exhibit higher resistance to the effects of ONOO^−^ on the behavior of capsid proteins and HBGA-binding under the same conditions.

To conclude, free chlorine appears to affect GII.4 VLPs and GII.4 HuNoVs by altering VP1 dimer interactions. Both are sensitive to critical concentrations of 9,531 and 19 μM for GII.4 VLPs and GII.4 HuNoVs, respectively, leading to the release of VP1 monomers and, to a lesser extent, the formation of some aggregates through cross-links. In both particles, the dissociation of VP1 dimers stops all interaction with HBGAs. The application of ONOO^−^ had different effects on GII.4 VLPs and GII.4 HuNoVs. ONOO^−^ treatment had less effect on GII.4 HuNoV capsids than on GII.4 VLPs. In particular, it had less effect on the dissociation of VP1 dimers and on interactions with HBGAs, even at a concentration capable of dissociating GII.4 VLPs. Further investigations could be performed using other strategies (e.g., fourier transform infraRed spectroscopy, FTIR) to confirm the influence of oxidant concentration on VLPs, especially on the distribution of VP1 monomers, dimers, and aggregates by SDS-PAGE analysis.

## Data Availability Statement

The original contributions presented in the study are included in the article/Supplementary Material, further inquiries can be directed to the corresponding author.

## Author Contributions

CG and NB designed the study. MC performed the experiments. GBa performed MALDI-TOF experiments and analyzed the results. MC, GBa and CG analyzed the data and prepared the Figures. MC, GBa, NB, and CG wrote the manuscript. MR performed statistical analysis. AR and GBe provided highly concentrated and purified GII.4 VLP suspensions. DM, NB, and CG were involved in the intellectual development of the project and the acquisition of funding. All authors contributed to the article and approved the submitted version.

### Conflict of Interest

The authors declare that the research was conducted in the absence of any commercial or financial relationships that could be construed as a potential conflict of interest.

## Footnotes

^1^https://web.expasy.org/peptide_mass/
