# Supplementary material for: Free Chlorine and Peroxynitrite Alter the Capsid Structure of Human Norovirus GII.4 and Its Capacity to Bind Histo-Blood Group Antigens
Source: Front Microbiol. 2021 Apr 13;12:662764. doi: 10.3389/fmicb.2021.662764 (PMC8076513; doi:10.3389/fmicb.2021.662764)
Supplement: Supplementary file 1 [file Data_Sheet_1.docx]

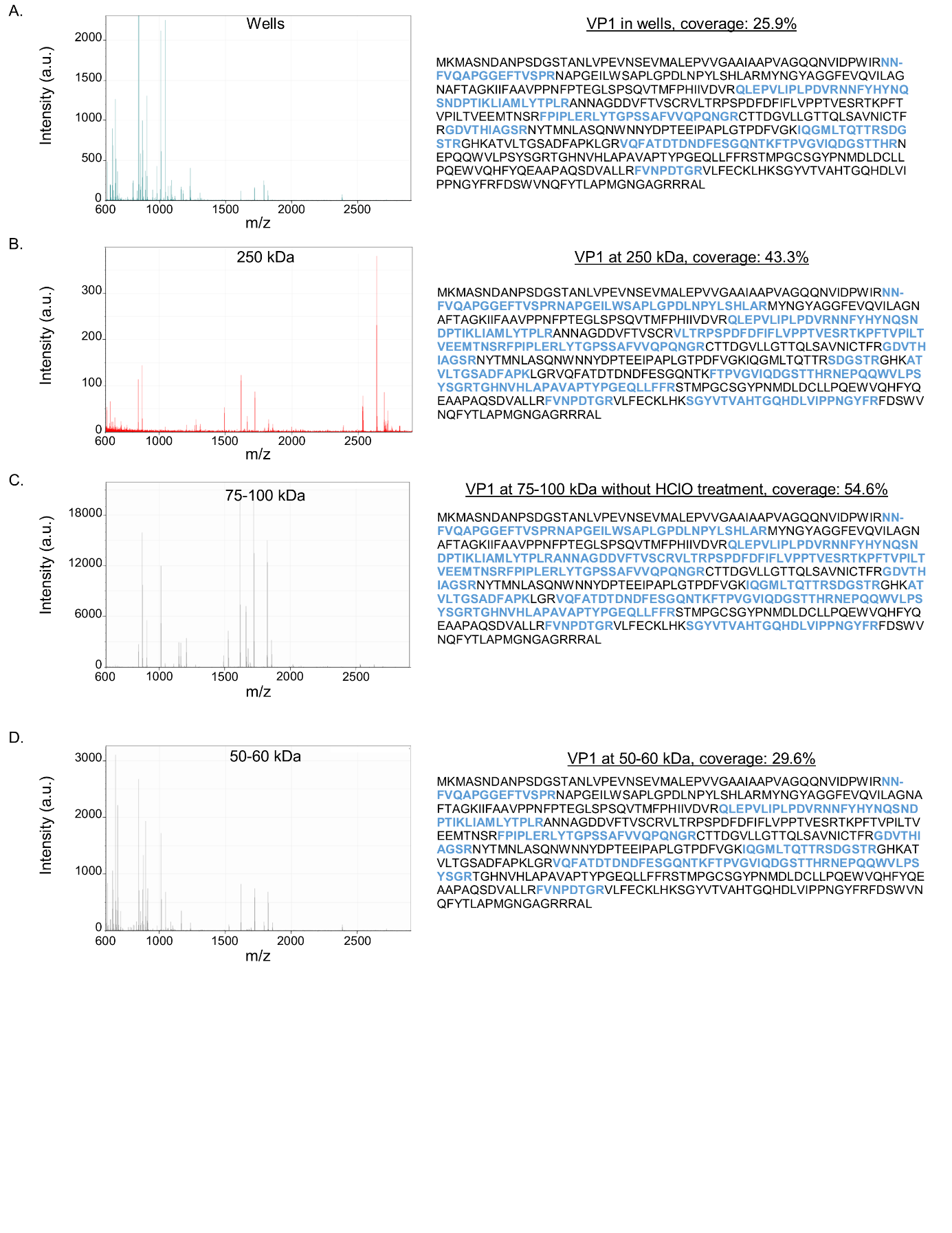


**Supplementary Figure S1: MALDI-TOF MS of GII.4 VLP proteins from SDS-PAGE.** The left panel displays the spectrum of GII.4 VLPs treated by 9,531 µM of free chlorine, corresponding to the protein bands in the well of the stacking gel by SDS-PAGE after digestion by trypsin **(A)**. The left panel displays the spectrum of GII.4 VLPs treated by 9,531 µM of free chlorine, corresponding to the protein bands at ~250 kDa by SDS-PAGE after digestion by trypsin **(B)**. The left panel displays the spectrum of native GII.4 VLPs, corresponding to the protein bands at ~75-100 kDa by SDS-PAGE after digestion by trypsin **(C)**. The left panel displays the spectrum of GII.4 VLPs treated by 9,531 µM of free chlorine, corresponding to the protein bands at ~50-60 kDa by SDS-PAGE after digestion by trypsin **(D)**. All right panels display the amino acid sequence of theVP1 of GII.4 VLPs. Letters in blue and bold correspond to peptides recovered from the peptide mass fingerprint as obtained in the respective left panels. Carbamidomethylation and methionine modifications were taken in account for peptide mass fingerprinting. The data are representative of two independent experiments performed in separate days.


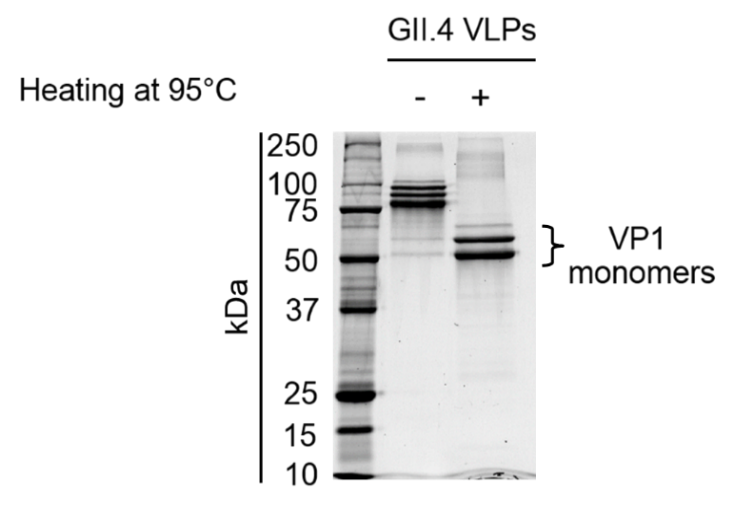


**Supplementary Figure S2: Dissociation of VP1 dimers in monomers after heat treatment**. “Heating at 95 °C” means that GII.4 VLPs were heated at 95 °C for 10 min before SDS-PAGE analysis. The “+” and “−” signs indicate whether the heat treatment was applied.**
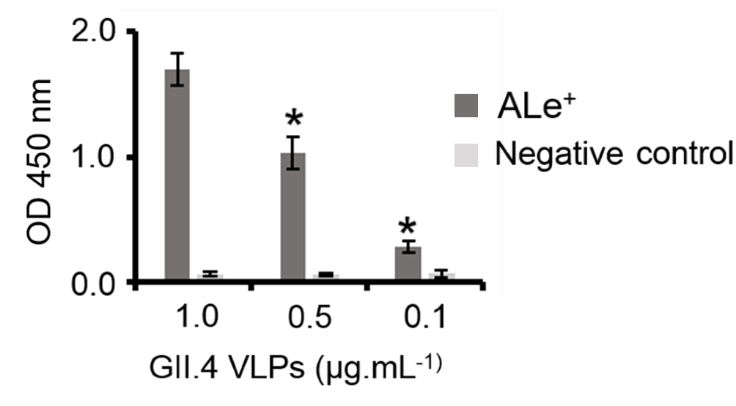
**

**Supplementary Figure S3: Sensitivity of HBGA-binding assays to GII.4 VLPs using ALe^+^ saliva.** Each data point corresponds to the mean OD_450_ values of duplicates from three independent experiments carried out in separated days. Negative controls represent the HBGA-binding to GII.4 VLPs using ALe^+^ saliva treated by sodium periodate. Error bars indicate standard deviations. *Unpaired Student’s *t*-test was used to compare groups with *p* < 0.001.

**
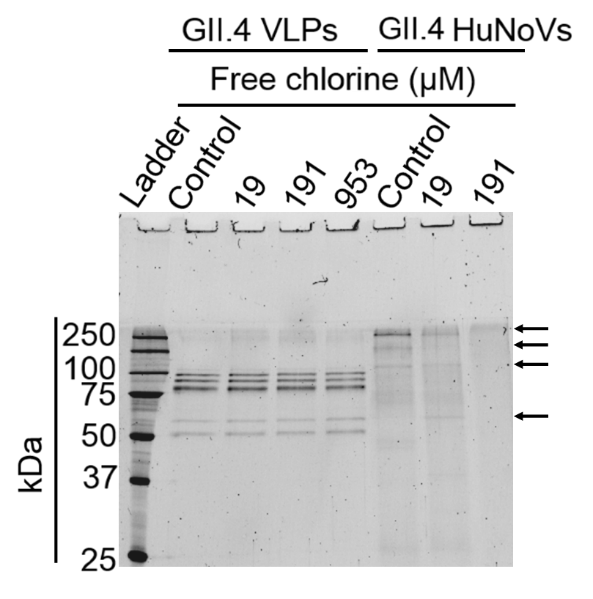
**

**Supplementary Figure S4: Different SDS-PAGE profiles between capsid proteins of GII.4 HuNoVs and GII.4 VLPs after free chlorine treatment in the same conditions.** GII.4 VLPs (20 µg.mL^-1^) and GII.4 HuNoVs (44 µg.mL^-1^) were analyzed by SDS-PAGE under denaturing conditions after treatment by free chlorine at 19, 191 and 953 µM without heat treatment. The control corresponds to native GII.4 VLPs and GII.4 HuNoVs. Black arrows indicated the main protein bands of GII.4 HuNoVs observable by SDS-PAGE under denaturing conditions.
